# Synthesis and Characterization of Self-Assembled Nanogels Made of Pullulan

**DOI:** 10.3390/ma4040601

**Published:** 2011-03-25

**Authors:** Sílvia A. Ferreira, Paulo J. G. Coutinho, Francisco M. Gama

**Affiliations:** 1 IBB-Institute for Biotechnology and Bioengineering, Centre for Biological Engineering, Minho University, Campus Gualtar 4710-057, Braga, Portugal; E-Mail: silviarmferreira@deb.uminho.pt; 2 Centre of Physics, Minho University, Campus Gualtar 4710-057, Braga, Portugal; E-Mail: pcoutinho@fisica.uminho.pt (P.C.)

**Keywords:** pullulan, synthetic nanogels, self-assembly, amphiphilic macromolecular micelles, Michael addition, hydrophobic domains

## Abstract

Self-assembled nanogels made of hydrophobized pullulan were obtained using a versatile, simple, reproducible and low-cost method. In a first reaction pullulan was modified with hydroxyethyl methacrylate or vinyl methacrylate, further modified in the second step with hydrophobic 1-hexadecanethiol, resulting as an amphiphilic material, which self-assembles in water via the hydrophobic interaction among alkyl chains. Structural features, size, shape, surface charge and stability of the nanogels were studied using hydrogen nuclear magnetic resonance, fluorescence spectroscopy, cryo-field emission scanning electron microscopy and dynamic light scattering. Above the critical aggregation concentration spherical polydisperse macromolecular micelles revealed long-term colloidal stability in aqueous medium, with a nearly neutral negative surface charge and mean hydrodynamic diameter in the range 100–400 nm, depending on the polymer degree of substitution. Good size stability was observed when nanogels were exposed to potential destabilizing pH conditions. While the size stability of the nanogel made of pullulan with vinyl methacrylate and more hydrophobic chains grafted was affected by the ionic strength and urea, nanogel made of pullulan with hydroxyethyl methacrylate and fewer hydrophobic chains grafted remained stable.

## 1. Introduction

Pullulan is a water soluble, linear, neutral extracellular biodegradable homopolysaccharide of glucose produced by the fungus *Aureobasidium pullulans* (*Pullularia pullulans*) [1,2,3,4]. Pullulan consists of maltotriosyl units connected by α-D-1,6-glycoside linkages [3,5]. Pullulan is extensively used in food, cosmetic and pharmaceutical industries because it is easily modifiable chemically, non-toxic, non-immunogenic, non-mutagenic, and non-carcinogenic [5,6]. Furthermore, pullulan has good mechanical properties and attractive functional properties, such as adhesiveness, film formability, and enzymatically-mediated degradability [7]. In the form of self-assembled nanogels, it has been shown to exhibit chaperon like activity, thus being a promising technique for protein refolding [8]. It has been studied as a blood-plasma expander and substitute [9]. Pullulan arose as a promising polymer for various biomedical applications [10], such as surface modification of polymeric materials to improve blood compatibility (bioinert surfaces) [11,12], for gene [13,14] and drug delivery [5,15,16,17,18,19], as a carrier for quantum dots for intracellular labeling to be used as a fluorescent probe for diagnostic bioimaging [20] and tissue engineering [21]. Self-assembled biotinylated pullulan acetate nanoparticles loading adriamycin were described as targeted anti-cancer drug delivery systems, internalized by HepG2 cells. The drug loading and release rate were accessed with a dialysis method [18]. Adriamycin loaded pullulan acetate/sulfonamide conjugate nanoparticles responding to tumor pH revealed pH-dependent cell interaction, internalization and cytotoxicity in *in vitro* studies using a breast tumor cell line (MCF-7). The drug loading profile was evaluated using a dialysis method [19]. Non-toxicity, efficient internalization and transfection *in vitro* of hydrogel pullulan nanoparticles encapsulating pBUDLacZ plasmid showed this system to be an efficient gene delivery carrier [14]. Pullulan potentially targets and accumulates in the liver because it is recognized by the asialoglycoprotein receptor expressed on the sinusoidal surface of the hepatocytes [22]. The asialoglycoprotein receptor was reported to be involved in pullulan receptor-mediated endocytosis [23].

The production of hydrophobically modified pullulan nanogels, using an approach similar to the one presented in this work, was achieved by other authors using cholesteryl group-bearing pullulan. The resulting nanogels were monodisperse, with a diameter of 20–30 nm and stable in water. Their size and density were controlled by the pullulan degree of substitution with cholesterol and the molecular weights of parent pullulan [24]. This nanogel was utilized in molecular complexation with bovine serum albumin (BSA) [25], insulin [26], lipase [27], human epidermal growth factor receptor 2 (HER-2) [28,29,30], interleukin-12 (IL-12) [31,32], among other therapeutic molecules, proving this system to be useful as a therapeutic delivery system. Self-assembled hydrogel nanoparticles of cholesterol-bearing pullulan spontaneously release insulin from the complex and thermal denaturation/aggregation were effectively suppressed upon complexation [26]. Cholesteryl group-bearing pullulan complexed with the truncated HER-2 protein, delivered a HER-2 oncoprotein containing an epitope peptide to the major histocompatibility complex class I pathway, and was able to induce CD8+ cytotoxic T lymphocytes against HER-2+ tumors and caused complete rejection of tumors. The results suggested this hydrophobized polysaccharide may help soluble proteins to induce cellular immunity with potential benefit in cancer prevention and cancer therapy [30]. The subcutaneous injection of cholesterol-bearing pullulan complexed with recombinant murine IL-12 led to a prolonged elevation of IL-12 concentration in the serum. Repetitive administrations of the complex induced drastic growth retardation of reestablished subcutaneous fibrosarcoma, without causing toxicity [31]. Raspberry-like assembly of nanogels encapsulated IL-12 efficiently (96%) and kept it stable in the presence of BSA (50 mg/mL) and showed high potential to maintain a high IL-12 level in plasma after subcutaneous injection in mice [32]. Cationic derivative, ethylenediamine group functionalization of cholesteryl group-bearing pullulan, was developed as an effective intracellular protein delivery system [33]. The same research group designed hybrid hydrogels with self-assembled nanogels as cross-linkers to achieve interaction with proteins and chaperone-like activity [32,34,35]

Nanogel formulations, described as potential drug and vaccine delivery systems, have the potential to modify the drug, gene, protein, peptide, oligosaccharide or immunogen profile and the ability to cross biological barriers, the biodistribution and pharmacokinetics, improving their efficacy and safety, as well as the patient compliance [36].

## 2. Results and Discussion

In the present work, hydrophobized pullulan was obtained with a two-step synthesis. The resultant self-assembled nanogels were characterized in terms of structure, size, shape, surface charge and stability by hydrogen nuclear magnetic resonance (^1^H NMR), fluorescence spectroscopy, cryo-field emission scanning electron microscopy (cryo-FESEM) and dynamic light scattering (DLS).

### 2.1. Synthesis of Pullulan-C_16_

**Scheme I:**
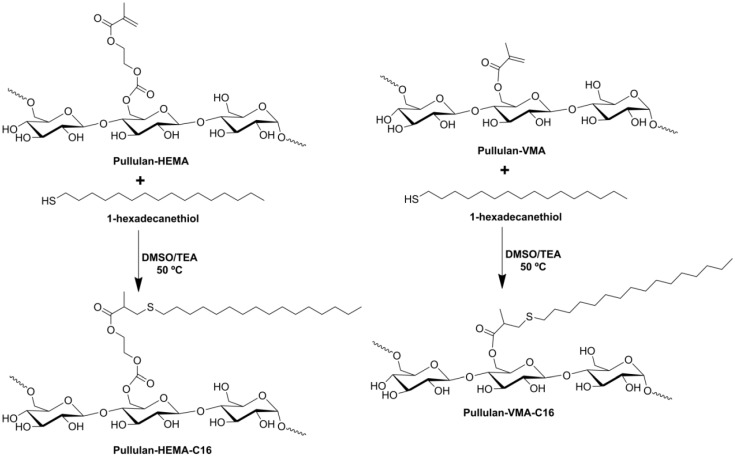
Synthesis of pullulan-C_16_.

**Figure 1:**
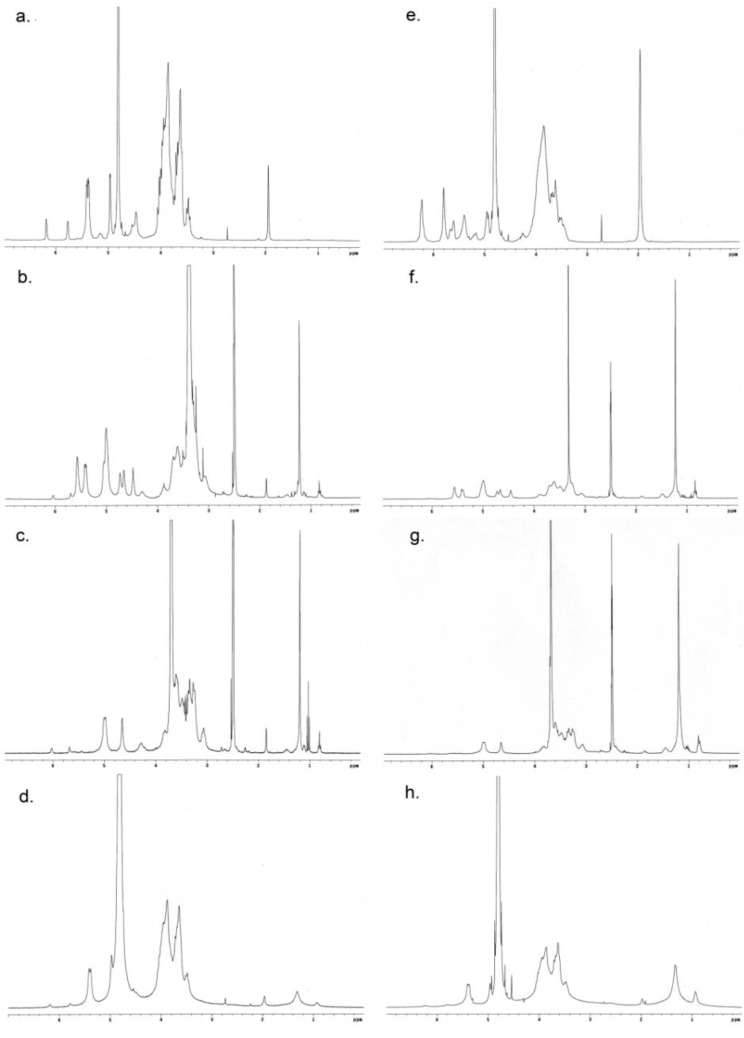
^1^H NMR spectra of (**a**) pullulan-HEMA and (**e**) pullulan-VMA in D_2_O. ^1^H NMR spectra of PHC_16_-5.6-1.3 and PVC_16_-10-7 in (**b**, **f**) DMSO-*d*_6_, (**c**, **g**) 10% D_2_O in DMSO-*d*_6_ and (**d**, **h**) D_2_O, respectively.

According to the literature and in the same way as other reported methacrylates, hydroxyethyl methacrylate (HEMA) and vinyl methacrylate (VMA) should be grafted on the ^6^C of the glucose residues [37]. Then, by the Michael addition mechanism, the thiol from 1-hexadecanethiol (C_16_) acting as a nucleophile reacts with grafted methacrylate (Scheme I). The success of the synthesis, purity, chemical structure and polymer degree of substitution of the reaction products were controlled using ^1^H NMR spectra in deuterium oxide (D_2_O) (Figure 1 and Table 1). Different independent batches of hydrophobized pullulan (pullulan-C_16_) with various degree of substitution with the methacrylated groups and hydrophobic alkyl chains (DS_HEMA_ or DS_VMA_ and DS_C16_, defined as the percentage of grafted HEMA or VMA or C_16_ moieties relative to the glucose residues, respectively), were synthesized by varying the molar ratios of methacrylate groups to glucose residues and the molar ratios of C_16_ to methacrylated groups. The synthetic procedure adopted proves to be versatile, simple and reproducible (Table 1).

**Table 1:** Characteristics of Pullulan-C_16_.

*t*DS_HEMA_ or *t*DS_VMA_*^a^*	DS_HEMA_ or DS_VMA_ *^b^*	*t*DS_C16_ *^c^*	DS_C16_*^d^*	DS_C16_/DS_HEMA_ or DS_C16_/DS_VMA_ *^e^*	Pullulan-C_16_ *^f^*
Pullulan-HEMA-C_16_					
20	5.6	120	1.3	23.2	PHC_16_-5.6-1.3
25	8	80	4.6	57.5	PHC_16_-8-4.6
		200	4.3	53.8	PHC_16_-8-4.3
40	10	80	1.2	12	PHC_16_-10-1.2
		200	5.9	59	PHC_16_-10-5.9
Pullulan-VMA-C_16_					
25	8.8	200	6	68.2	PVC_16_-8.8-6
50	10	200	7	70	PVC_16_-10-7

*^a^* Theoretical DS_HEMA_ or DS_VMA_ calculated as the molar ratio of HEMA or VMA to glucose residue (×100) in the reaction mixture. *^b^* Calculated from the ^1^H NMR spectra in D_2_O of pullulan-HEMA or pullulan-VMA in D_2_O with the equation (*I*_a_)/(*I*_H1_)×100, in which *I*_a_ is the average integral of the protons of the unsaturated carbons of the acrylate groups (around 6 ppm) [38,39] and *I*_H1_ is the integral of the anomeric protons (4.86, 5.28 and 5.30 ppm) [4,40]. *^c^* Theoretical DS_C16_ calculated as the molar ratio of C_16_ to methacrylated groups (×100) in the reaction mixture. *^d^* Calculated from the ^1^H NMR spectra of pullulan-C_16_ in D_2_O with the equation (7*X*)/(37*Y*)×100, in which *X* is the average integral corresponding to the protons from alkyl moieties (1.8–0.6 ppm) [41] and *Y* is the integral of all pullulan protons (3.3–4.0 ppm and 4.86, 5.28 and 5.30 ppm) [4,40]. *^e^* Obtained DS_C16_ relative to methacrylated groups calculated using the following equation: DS_C16_/DS_HEMA_ (×100) or DS_C16_/DS_VMA_ (×100)_._ *^f^* Pullulan-HEMA-SC_16_ synthesized: PHC_16_-DS_HEMA_-DS_C16_; or Pullulan-VMA-SC_16_ synthesized: PVC_16_-DS_VMA_-DS_C16._ The table presents the values (%) obtained in each set of conditions.

### 2.2. Self-assembly of Pullulan-C_16_

The self-assembly of amphiphilic pullulan-C_16_ in water was studied using ^1^H NMR and fluorescence spectroscopy. Analyzing the ^1^H NMR spectra of pullulan-C_16_ (Figure 1), it can be observed that while the mobility of the polysaccharide skeleton was maintained in environments of different polarity, the shape and width of the proton signals of the methyl (0.8 ppm) and methylene (1.1 ppm) groups of C_16_ depended on the polarity of the solvent used. In dimethyl sulfoxide-*d*_6_ (DMSO-*d*_6_), pullulan-C_16_ was soluble, and the C_16_ signals were sharp, as all hydrophobic chains were exposed to the solvent, having the same mobility (Figure 1b and 1f) [41]. Increasing the percentage of D_2_O in DMSO-*d*_6*,*_ the base of those signals broadened (Figure 1c and 1g). In pure D_2_O, a large broadening was obvious, which represents the superposition of peaks of chemically identical species, yet possessing various degrees of mobility (Figure 1d and 1h) [42]. These results give evidence that pullulan-C_16_ dispersed in water has part of the alkyl chains exposed to hydrophobic domains, while others might have been exposed to the hydrophilic solvent. Differences in the environment and/or mobility of the molecules thus explain the broad peak observed for the aliphatic protons. Therefore, pullulan-C_16_ nanogels are obtained upon self-assembly in water through the association of the hydrophobic alkyl chains in hydrophobic domains.

The critical aggregation concentration (cac) or critical micelle concentration (cmc) of pullulan-C_16_ was studied by fluorescence spectroscopy using hydrophobic dyes, Pyrene (Py) [43,44] and Nile red (NR) [45], whose solubility and fluorescence are weak in water but high in hydrophobic environments.

**Figure 2:**
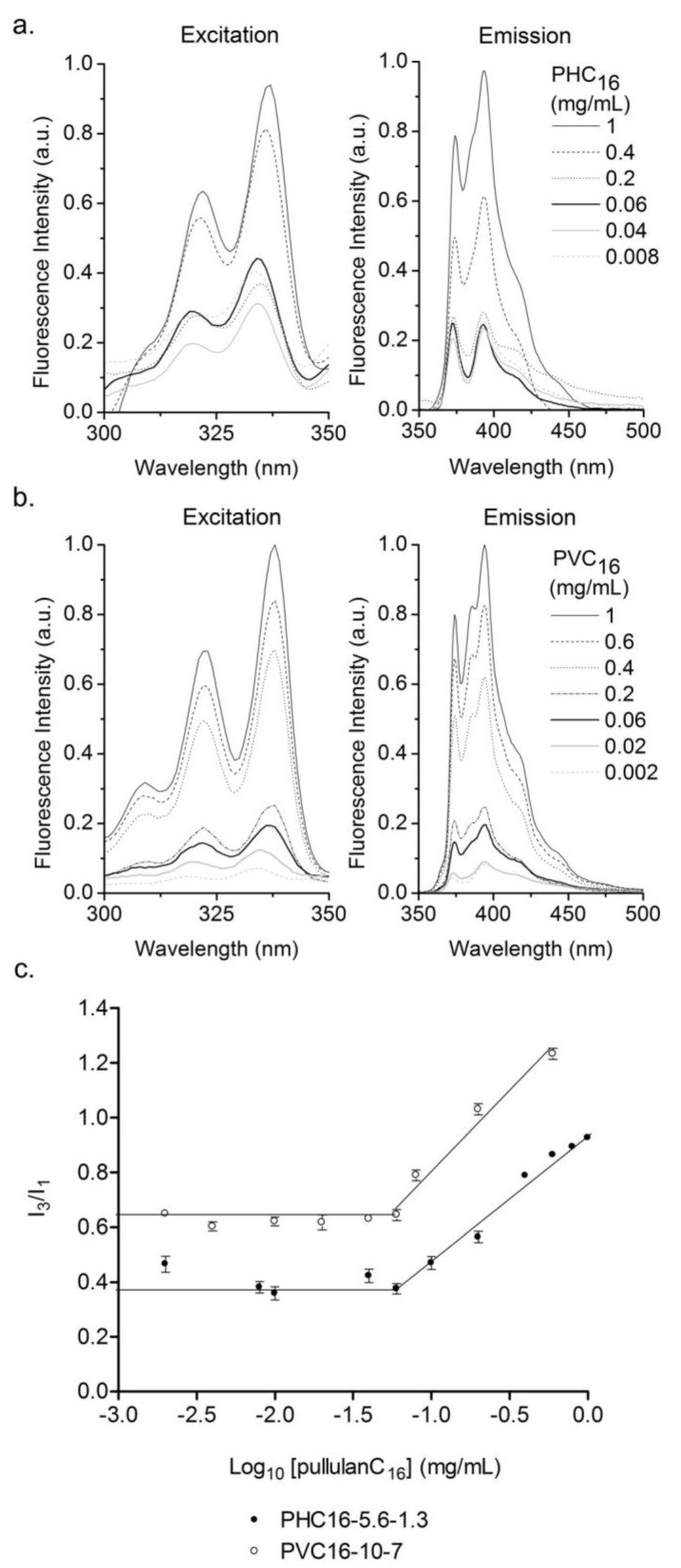
Determination of the critical aggregation concentration (cac) of pullulan-C_16_ using fluorescence excitation (λ_em_ = 390 nm) and emission (λ_ex_ = 339 nm) spectra of Py (6 × 10^−7^ M) in the pullulan-C_16_/water system as a function of the (**a**) PHC_16_-5.6-1.3 and (**b**) PVC_16_-10-7 concentration; (**c**) Intensity ratio I_3_/I_1_ as a function of the pullulan-C_16_ concentration. The cac obtained for both materials was 0.06 mg/mL.

The intensity of Py increased with increasing concentrations of pullulan-C_16_, and a red shift occurred in the excitation spectra (Figure 2a, 2b). Above cac, in the emission spectra (Figure 2a, 2b), some bands in the 450 nm region associated to the presence of Py dimers are detected in pullulan-C_16_, suggesting high water penetration into the nanogel, which is in agreement with the ^1^H NMR measurements. The intensity ratio of the third and first vibrational bands, I_3_/I_1,_ rapidly augmented above the cac, which was 0.06 mg/mL for PHC_16_-5.6-1.3 and for PVC_16_-10-7. This transition of intensity translated the transference of Py to a less polar and hydrophobic domain that was coincident to the onset of supramolecular formation of pullulan-C_16_ nanogels (Figure 2c). A lower I_3_/I_1_ ratio obtained for PHC_16_-5.6-1.3 indicates the location of Py in a more hydrophilic environment, while a higher I_3_/I_1_ ratio for PVC_16_-10-7 indicates the location of Py in a more hydrophobic environment (Figure 2c) [43]. This is confirmed by a better defined vibronic structure of Py emission in the case of PVC_16_-10-7. Surprisingly, the resulting cac is the same for both nanogels despite their different DS_C16_ relative to methacrylated groups (70% for PVC_16_-10-7 and 23% for PHC_16_-5.6-1.3).

**Figure 3:**
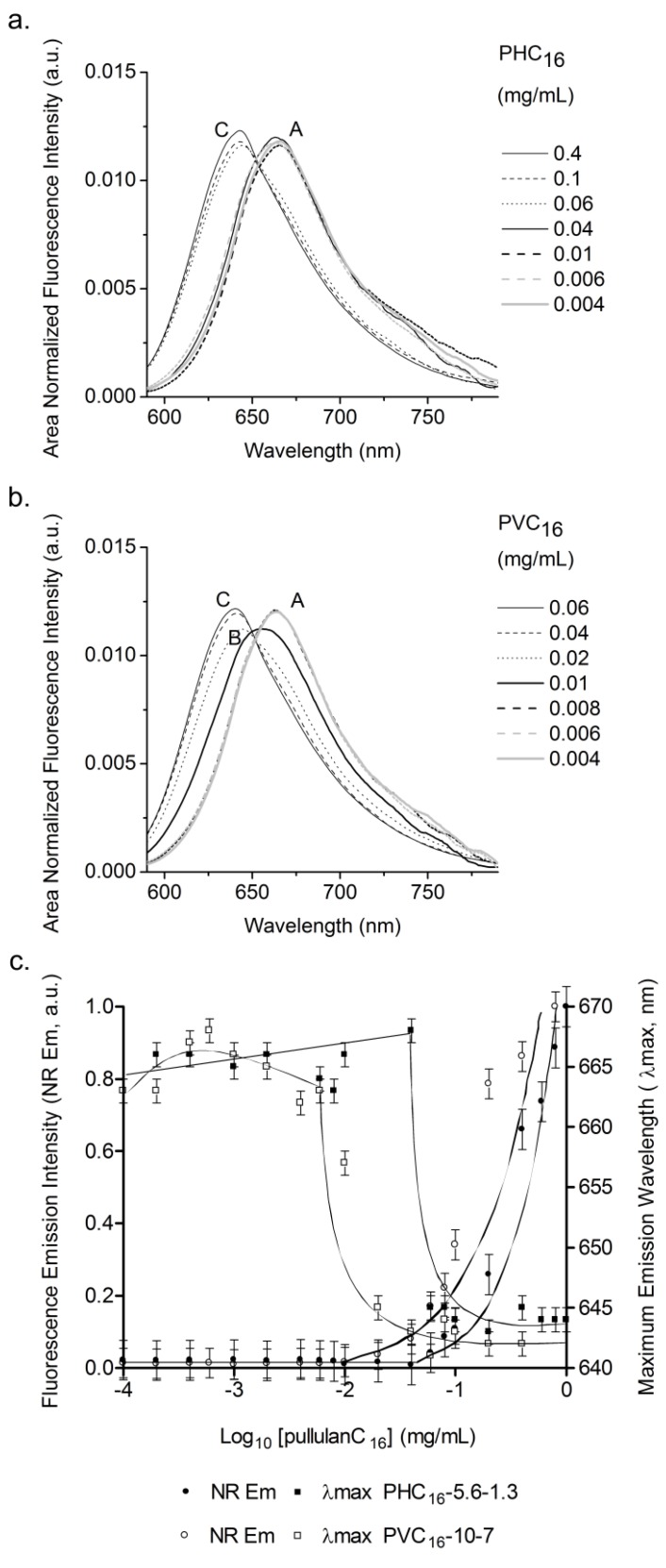
Determination of the cac of pullulan-C_16_ using area normalized fluorescence emission (λ_ex_ = 570 nm) spectra of NR (2 × 10^−7^ M) in the pullulan-C_16_/water system as a function of (**a**) PHC_16_-5.6-1.3 and (**b**) PVC_16_-10-7 concentration; (**c**) area normalized fluorescence emission intensity and position of maximum emission wavelength of Nile red (NR) in the pullulan-C_16_/water system as a function of pullulan-C_16_ concentration. The cac obtained for PHC_16_-5.6-1.3 was 0.04 mg/mL and for PVC_16_-10-7 was 0.01 mg/mL.

The area-normalized fluorescence emission intensity of NR was constant, without any shift in the maximum emission wavelength, for lower concentrations of pullulan-C_16_ because individual molecules exist as premicelles in aqueous environment (Figure 3; zone A). In contrast, for concentrations greater than the cac, fluorescence intensity increased and the maximum emission wavelength was blue-shifted due to the transfer of NR to the hydrophobic domains of the nanogels. The resultant cac was 0.04 mg/mL and 0.01 mg/mL for PHC_16_-5.6-1.3 and PVC_16_-10-7, respectively (Figure 3). This variation is consistent with the C_16_ loading of the studied pullulan nanogels as higher hydrophobicity results in lower cac. The PVC_16_-10-7 hydrophobic domains are dissimilar to those present in a typical surfactant system and have two types of hydration levels (Figure 3b; zones B and C), while in PHC_16_-5.6-1.3 only a type of hydrophobic domains is observed (Figure 3a; zone C). This observation shows a slight dependence of the formed hydrophobic domains on the type of linker used (HEMA or VMA).

In the case of PHC_16_-5.6-1.3, the determined cac values are similar for both fluorescent probes. But that is not the case for PVC_16_-10-7. This is explainable by the fact that as Py molecules already start at a low hydrated pre-micellar environment they are unable to detect the micellar domains of type B, which have higher hydration levels than those domains of type C. For the last ones there is a sufficient variation of hydration level that can be detected by Py I_3_/I_1_ ratio resulting in a cac value above the real one. We thus conclude that NR is a more sensitive fluorescence probe as it was able to follow all the variations in hydration level that occurred in the self-aggregation process of PVC_16_-10-7. For PHC_16_-5.6-1.3 the absence of B type micellar domains and the higher hydration of the premicellar environment, also seen in NR emission in zone A, allowed compatible determinations of cac for both probes.

As pullulan-C_16_ concentration augments above the cac, more hydrophobic domains are formed, solubilizing more Py and NR, which consequently increases the fluorescence detected, not occurring the typical second plateau (Figure 2c, Figure 3c). The highest concentration of pullulan-C_16_ used was insufficient to enclose all of the hydrophobic dyes—this might be caused by the continued redistribution of Py and NR molecules to the less hydrated hydrophobic domains and by the formation of Py dimers in the hydrophobic domains with greater hydration level.

### 2.3. Characterization of Pullulan-C_16_ Nanogels

#### 2.3.1. Size and shape

**Figure 4:**
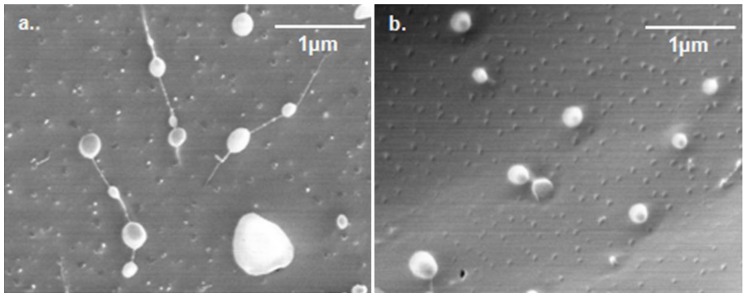
Cryo-FESEM negatively stained micrographs (magnification 30000×) of (**a**) PHC_16_-5.6-1.3 and (**b**) PVC_16_-10-7.

The hydrophobic forces that sequester the hydrophobic chains in the core and the excluded volume repulsion between the chains mostly establish the micellar size [46]. The pullulan-C_16_ nanogels appeared spherical in cryo-FESEM micrographs, with a large size distribution in the range of 100–700 nm for PHC_16_-5.6-1.3 and 200–300 nm for PVC_16_-10-7 (Figure 4).

#### 2.3.2. Storage

**Figure 5:**
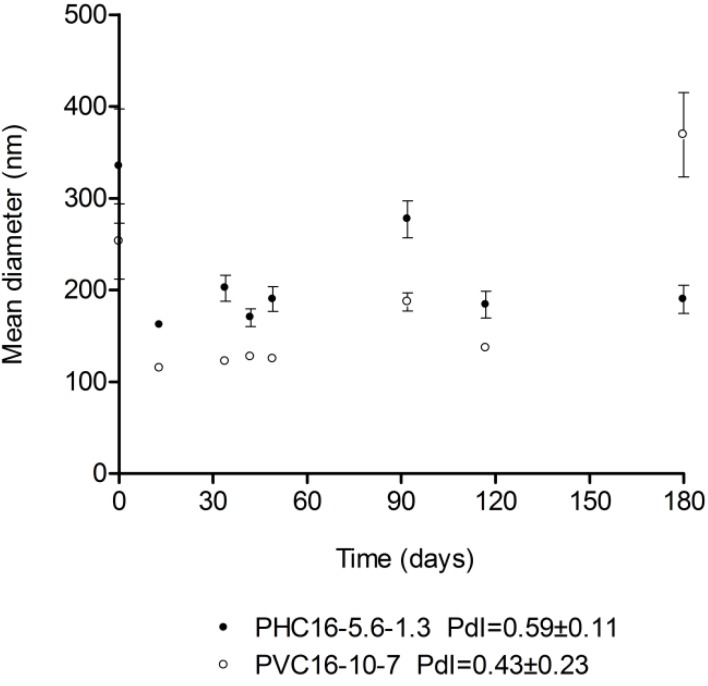
Size of pullulan-C_16_ water dispersions (1 mg/mL) over a 6 month storage period at room temperature (25 °C). Size was measured periodically in dynamic light scattering (DLS) (mean ± S.D., n = 10).

The mean hydrodynamic diameter obtained using DLS for pullulan-C_16_ nanogels dispersed in ultrapure water oscillated between 162 nm and 335 nm for PHC_16_-5.6-1.3 and between 115 nm and 369 nm for PVC_16_-10-7, over a six month storage period at room temperature (25 °C). Both materials exhibited fairly high polydispersity, with an average pdI of 0.59 ± 0.11 for PHC_16_-5.6-1.3 and 0.43 ± 0.23 for PVC_16_-10-7, which means that there may be macromolecular micelles with a distribution of sizes and shapes, as also revealed by the cryo-FESEM micrographs (Figure 5).

#### 2.3.3. Effect of the Concentration of Pullulan-C_16_

**Figure 6:**
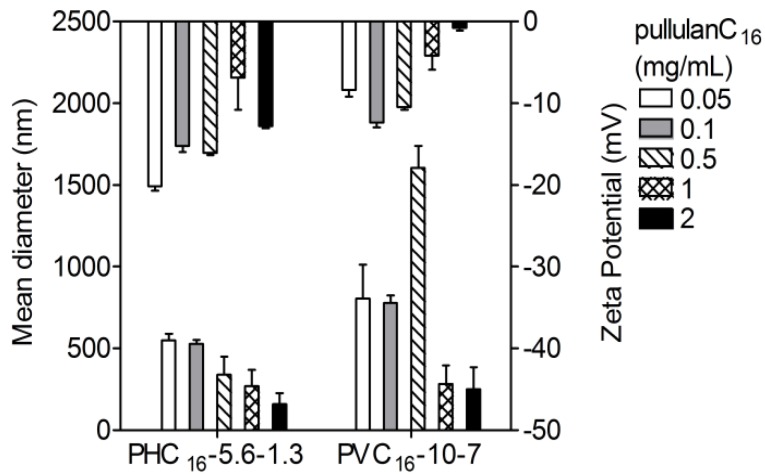
Influence of concentration on the size and zeta potential of pullulan-C_16_ nanogels (0.05–2 mg/mL) measured at 37 °C in DLS (mean ± S.D., n = 3).

The mean hydrodynamic diameter tended to be much larger for lower concentrations of pullulan-C_16_, especially when closer to the cac. It appears that, for higher concentrations of the polymer, the remaining solvent is gradually released from the hydrophobic core, resulting in a decrease in size. In contrast, occasionally exposed hydrophobic domains within a less mobile shell formed by hydrophilic chains may originate secondary aggregation enlarging the resultant macromolecular micelles [46]. The zeta-potential values were always negative and close to zero, never lower than −20 mV. Once zeta potential approaches zero, electrostatic repulsion becomes small compared to the ever-present Van der Waals attraction. In these conditions, eventually, instability may arise, causing aggregation followed by sedimentation and phase separation. However, the pullulan-C_16_ nanogels preserved their nanosize with the exception of PVC_16_-10-7 at 0.5 mg/mL that formed aggregates out of the nanoscale (Figure 6).

#### 2.3.4. Effect of Urea

**Figure 7:**
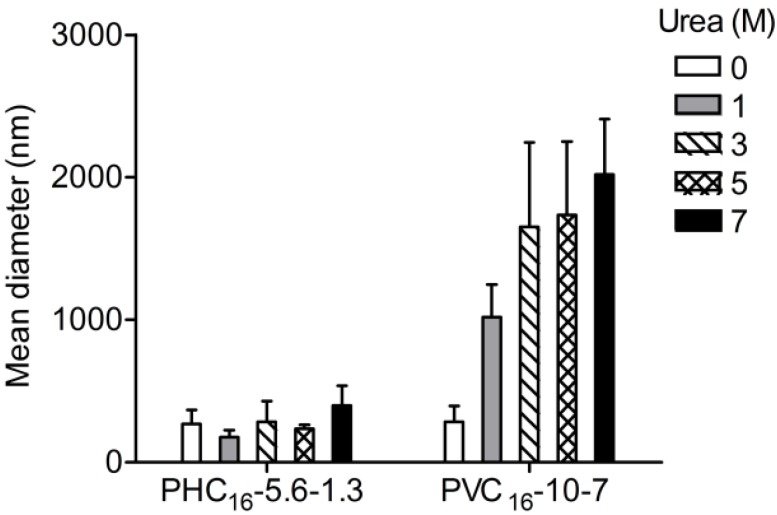
Influence of urea (0–7 M) on the size of pullulan-C_16_ nanogels (1 mg/mL) measured at 37 °C in DLS (mean ± S.D., n = 3).

Urea is known for its ability to break intramolecular hydrogen bonds and to destabilize hydrophobic domains [47,48]. Urea and its derivatives are very efficient as modifiers of the aqueous solution properties participating at the level of the micellar solvation layer because it enhances the polarity and the hydrophilic character of water. An increased accessibility from the aqueous phase at higher urea concentrations could result in a stronger solvation of the polar groups in micellar aggregates by urea−water mixture than water alone. Urea is related to the enhancement of the solubility of hydrocarbon tails favoring their solvation and to the weakening of the hydrophobic interactions responsible for the formation and maintenance of the micellar assembly in aqueous solution. The action of urea on micellization depends on the way in which solvation occurs in a specific micellar system [49]. The results obtained show that urea did not affect the nanogel size of PHC_16_-5.6-1.3. In contrast, urea caused concentration dependent destabilization of PVC_16_-10-7, affecting the self-assembly of this amphiphilic system in water, leading to the formation of larger aggregates out of the nanoscale (Figure 7). Destabilization of PVC_16_-10-7, resulting in higher particle size, may be tentatively assigned to improved solvation of the hydrophobic domains. This possibility is supported by the fact that PVC_16_ has a higher substitution degree than PHC_16_ (DS_C16_ of 7 *vs.* 1.3).

#### 2.3.5. Effect of Ionic Strength

**Figure 8:**
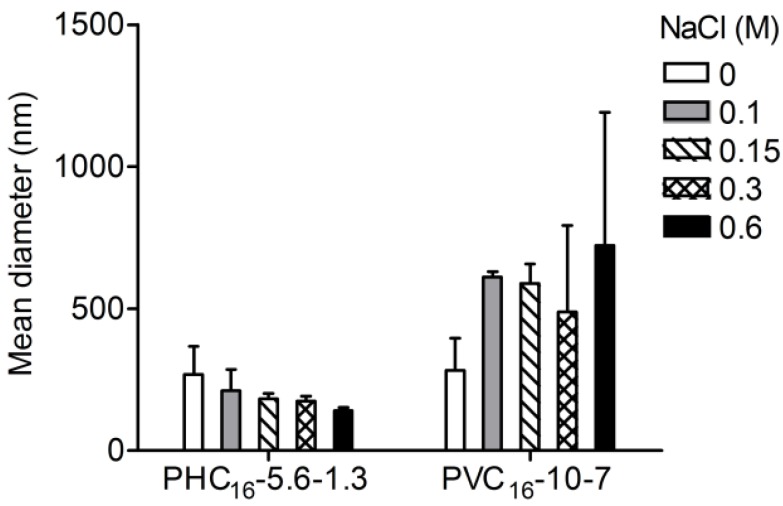
Influence of NaCl (0–0.6 M) on the size of pullulan-C_16_ nanogels (1 mg/mL) measured at 37°C in DLS (mean ± S.D., n = 3).

Colloidal stability might be compromised in the absence of an electrostatic barrier. The addition of enough quantity of salt neutralizes the surface charge of the micelles in dispersion and compresses the surface double layer, facilitating the colloidal aggregation. Without the repulsive forces that keep macromolecular micelles separate, coagulation might occur due to attractive Van der Waals forces. Compared to salt-free pullulan-C_16_ colloidal dispersion, while PHC_16_-5.6-1.3 denoted stability, PVC_16_-10-7 nanogel was larger as the ionic strength increased with increasing concentrations of NaCl (Figure 8).

#### 2.3.6. Effect of pH

**Figure 9:**
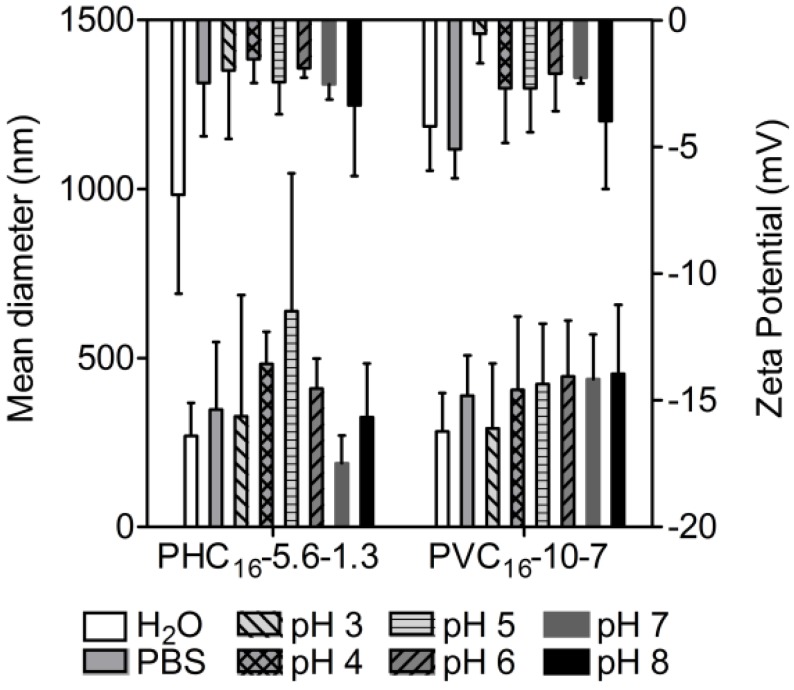
Influence of pH on the size and zeta potential of pullulan-C_16_ nanogels measured at 37 ºC in DLS (mean ± S.D., n = 3).

Size distributions and zeta potential of pullulan-C_16_ as a function of pH, using phosphate-citrate buffer (pH 2.2–8.0), were compared to values obtained in water and PBS. The mean hydrodynamic diameter values obtained either for PHC_16_-5.6-1.3 or PVC_16_-10-7 were similar in the range of pH studied. The size stability, in the range of pH studied, demonstrates that the organization of hydrophobic alkyl chains, in hydrophobic domains with low water content, protect the amphiphilic molecules from the hydrolysis of the carbonate ester at alkaline pH and from the hydrolysis of the methacrylate ester at low pH [50]. For both materials, small negative values of zeta potential were obtained indicating little repulsion between macromolecular micelles to prevent aggregation. However, even with zeta potential close to zero, particles denoted only slight instability in the nanoscale (Figure 9). The nearly neutral charge is valuable for *in vivo* use, since large positively charged materials cause non-specific cell sticking, while large negatively charged materials are efficiently taken up by scavenger endothelial cells or “professional pinocytes” found in the liver, which results in a rapid clearance from the blood [51].

Pullulan-based nanogels synthesized and characterized in this work have high water content, tunable size, interior network for possible incorporation of therapeutics, and large surface area for potential multivalent bioconjugation with cell-targeting ligands such as protein, peptides and antibodies. With these characteristics, described nanogels might be useful as polymeric carriers for therapeutic targeted delivery.

In our laboratory several nanogels are being developed, using different polysaccharides: dextrin, mannan, hyaluronic acid, glycolchitosan. The use of different polysaccharides allows the production of nanogels bearing different surface properties, namely size, charge and bioactivity. Among the applications envisaged for these materials, 1) the delivery of therapeutic proteins and of poorly water soluble pharmaceuticals, 2) vaccination, and 3) delivery of nucleic acid therapeutics are being developed. The comprehensive characterization of several nanogels provides a platform for the development of more sophisticated materials, with ability to perform as delivery systems. Recent results in our laboratory demonstrate the potential of dextrin nanogels for the delivery of cytokines, namely IL-10 [52]; the association of the nanogels with injectable hydrogels is also a promising field of application of the self-assembled nanogels, allowing the incorporation of hydrophobic molecules in the highly hydrated environment of hydrogels. Ongoing work addresses the study of biodistribution and drainage of nanogels to the lymphatic nodes. Preliminary results using radioactively labeled nanogels and immunohistochemical analysis of the lymphatic nodes confirm the ability of the nanogels to reach the nodes, internalized in phagocytic cells. The use of mannan opens interesting possibilities concerning the use of the nanogels for vaccination purposes, acting as a delivery system and as an adjuvant. Self-assembled nanogels are thus very promising materials that bring together the essential requisites of biocompatibility and performance.

## 3. Experimental Section

### 3.1. Materials

CDI-activated hydroxyethyl methacrylate (HEMA-CI) was produced as described elsewhere [38]. Pullulan (Mw = 100,000 g/mol), vinyl methacrylate (VMA), dimethyl sulfoxide (DMSO), 4-(N,N-dimethylamino)pyridine (DMAP), triethylamine (TEA), 1-hexadecanethiol (C_16_), deuterium oxide (D_2_O), dimethyl sulfoxide-*d*_6_ (DMSO-*d*_6_), pyrene (Py), 9-(diethylamino)-5H-benzo[α]phenoxazin-5-one (Nile red, NR) were purchased from Sigma-Aldrich. Pyrene was purified by appropriate recrystallization from absolute ethanol. Phosphotungstic acid was purchased from Riedel-de Haën. Regenerated cellulose tubular membranes, with a 12000−14000 nominal MWCO, were obtained from Membrane Filtration Products. Water was purified with a Milli-Q system (Millipore) with resistivity equal to 18.2 MΩ.cm. Other organic and inorganic chemicals were purchased from Sigma-Aldrich and used without further purification.

### 3.2. Synthesis of Amphiphilic Pullulan-C_16_

Hydroxyethyl methacrylate-derivatized pullulan (pullulan-HEMA) was prepared as described by Van Dijk-Wolthuis *et al.* [38]. Briefly, pullulan was dissolved in dry DMSO in a nitrogen atmosphere with different calculated amounts of HEMA-CI, resulting in 0.20, 0.25 and 0.4 molar ratios of HEMA-CI to glucose residues. The reaction catalyzed by DMAP (2 mol equiv to HEMA-CI) was allowed to proceed and the mixture was stirred at room temperature for 4 days. The reaction was terminated with concentrated HCl (2% v/v), which neutralized DMAP and imidazole. The mixture was then dialyzed against frequently changed distilled water at 4 °C for 3 days. After being lyophilized, pullulan-HEMA resulted as a white fluffy product, which was stored at −20 °C.

Vinyl methacrylated pullulan (pullulan-VMA) was synthesized by transesterification of pullulan with VMA, overall as described by Ferreira *et al.* [39] but without enzymes [53]. Briefly, pullulan was dissolved in dry DMSO, with calculated amounts of VMA resulting in 0.25 and 0.5 molar ratios of VMA to glucose residues. After stirring at 50 ºC for 2 days, the resulting mixture was dialyzed for 3 days against frequently changed distilled water, at room temperature (~25 ºC). Each sample of modified pullulan after being lyophilized resulted as a white fluffy product that was stored at room temperature.

Finally, the amphiphilic molecules pullulan-HEMA-C_16_ (PHC_16_) and pullulan-VMA-C_16_ (PVC_16_) were produced as described elsewhere [41]. In brief, Pullulan-HEMA or Pullulan-VMA reacted in dry DMSO (equivalent HEMA or VMA = 0.03 M) with C_16_. The reaction was catalyzed by TEA in a 2 molar ratio of TEA to HEMA or VMA. After stirring for 3.5 days at 50 ºC, the resulting mixture was dialyzed, lyophilized and stored as described above.

### 3.3. Characterization of pullulan-C_16_ nanogels

#### 3.3.1. ^1^H NMR Spectroscopy

Lyophilized reaction products were dispersed in D_2_O (5 mg/mL). The pullulan-C_16_ was also dispersed in DMSO-*d_6_* and in 10% D_2_O in DMSO-*d*_6_ (5 mg/mL). Samples were stirred overnight at 50 ºC to obtain a clear dispersion, which was transferred to 5 mm NMR tubes. One-dimensional ^1^H NMR measurements were performed in a Varian Unity Plus 300 spectrometer operating at 299.94 MHz. One-dimensional ^1^H NMR spectra were recorded at 298 K with 256 scans, a spectral width of 5000 Hz, a relaxation delay of 1 s between scans, and an acquisition time of 2.8 s.

#### 3.3.2. Fluorescence Spectroscopy

The cac of the pullulan-C_16_ was fluorometrically investigated using hydrophobic guest molecules, such as Py and NR. The fluorescence intensity change of these guest molecules was calculated as a function of the pullulan-C_16_ concentration. Briefly, lyophilized pullulan-C_16_ was dispersed in ultrapure water (1 mg/mL) with stirring for 3–5 days at 50 °C. Consecutive dilutions of 1mL of each sample were prepared in ultrapure water. In the case of Py, a volume of 5 μL of a 1.2 × 10^−4^ M Py stock solution in ethanol was added, giving a constant concentration of 6 × 10^−7^ M in 0.5 % ethanol/water for all Py fluorescence measurements. In case of NR, a volume of 5μL of a 4 × 10^−5^ M NR stock solution in ethanol was then added, giving a constant concentration of 2 × 10^−7^ M in 0.5 % ethanol/water for all NR fluorescence measurements. The samples were stirred overnight. Fluorescence measurements were performed with a Spex Fluorolog 3 spectrofluorimeter, at room temperature. The slit width was set at 5 nm for excitation and 5 nm for emission. All spectra were corrected for the instrumental response of the system. The signal obtained for each sample was subtracted with the signal obtained with negative control, which corresponded to pullulan derivatives at exactly the same experimental conditions but without the guest NR or Py molecules. The cac was calculated using both the Py fluorescence intensity ratio of the third (384–385 nm) and first vibrational bands (372–374 nm) (I_3_/I_1_) of the emission spectra (λ_ex_ = 339 nm) and the maximum emission intensity of NR (λ_ex_ = 570 nm) in the pullulan-C_16_/water system as a function of pullulan-C_16_ concentration; in both cases, the cac was estimated as the interception of two trend lines.

#### 3.3.3. Cryo-FESEM

Each colloidal dispersion of pullulan-C_16_ was prepared with stirring of the lyophilized pullulan-C_16_ in ultrapure water for 3–5 days at 50 °C (1 mg/mL) followed by filtration (pore size 0.45 μm), with insignificant material lost, as confirmed with the phenol-sulfuric acid method, using glucose as standard [54]. The colloidal dispersions were concentrated by ultrafiltration (Amicon Ultra-4 Centrifugal Filter Units, cutoff molecular weight 1 × 10^5^) and negatively stained with phosphotungstic acid (0.01% w/v). Samples were placed into brass rivets, plunged frozen into slush nitrogen at −200 ºC and transferred to the cryo stage (Gatan, Alto 2500, U.K.) of an electronic microscope (SEM/EDS: FESEM JEOL JSM6301F/Oxford Inca Energy 350). Each sample was fractured on the cryo stage with a knife. Once in the microscope, sublimation of ice was carried out in the cryo chamber for 10 min at −95 ºC, allowing the exposure of the nanogel particles. The samples were sputter coated with gold and palladium at −140 ºC, using an accelerating voltage of 10 kV. The antipollutant of copper covers and protects the sample. The samples were observed at −140 ºC at 15 kV. The solvent used in the preparation of the samples (water and phosphotungstic acid) was also observed as a negative control.

#### 3.3.4. DLS

The size distribution and zeta potential measurements for each colloidal dispersion, prepared as described above for cryo-FESEM, were performed in a Malvern Zetasizer NANO ZS (Malvern Instruments Limited, U.K.) using a He-Ne laser wavelength of 633 nm, a detector angle of 173° and a refractive index of 1.33.

*Size*. For each sample (1 mL), the polydispersity index (pdI) and z-average diameter, which corresponds to the mean hydrodynamic diameter, were evaluated in 10 repeated measurements performed periodically during 6 months of storage in a polystyrene cell at 25 °C. The size distribution of each sample dispersed in ultrapure water (0.05–2 mg/mL), phosphate-buffered saline (PBS 1x, pH 7.4), phosphate-citrate buffer (pH 2.2–8.0), NaCl (0–0.6 M) or in urea (0–7 M) was executed at 37 °C in three independent experiments, three repeated measurements being performed in each one.

*Zeta Potential.* Each sample dispersed in ultrapure water (0.05–2 mg/mL), phosphate buffered saline (PBS 1x, pH 7.4) or in phosphate-citrate buffer (pH 2.2–8.0) was analyzed at 37 °C in a folded capillary cell. The zeta potential values reported were calculated using the Smoluchowski equation with three independent experiments, three repeated measurements being performed in each one.

## 4. Conclusions

Hydrophobized pullulan nanogels were designed with a versatile, simple, reproducible and low-cost method. Above the cac, upon self-assembly in water, spherical polydisperse macromolecular micelles revealed long-term size stability in aqueous medium, with a nearly neutral negative surface charge and mean hydrodynamic diameter in the range 162–335 nm for PHC_16_-5.6-1.3 and 115–369 nm for PVC_16_-10-7. Size and zeta potential stability of pullulanC_16_ nanogels was maintained when exposed to potential destabilizing conditions of pH. While the size stability of the nanogel made of VMA with C_16_ grafted, PVC_16_-10-7, was affected by the ionic strength and urea, nanogel made of pullulan with HEMA and fewer C_16_ grafted, PHC_16_-5.6-1.3, remained more stable.

Pullulan-based nanogels have tunable size, high water content, interior network for possible incorporation of therapeutics, and large surface area for potential multivalent bioconjugation with cell-targeting ligands. With these characteristics, described nanogels might be useful as polymeric carriers for therapeutic targeted delivery. Further work is required to study molecular complexation, functionality and biocompatibility of these novel promising nanogels as drug and vaccine delivery systems.
